# Interferon-α (IFN-α) suppresses HTLV-1 gene expression and cell cycling, while IFN-α combined with zidovudin induces p53 signaling and apoptosis in HTLV-1-infected cells

**DOI:** 10.1186/1742-4690-10-52

**Published:** 2013-05-20

**Authors:** Shuichi Kinpara, Mami Kijiyama, Ayako Takamori, Atsuhiko Hasegawa, Amane Sasada, Takao Masuda, Yuetsu Tanaka, Atae Utsunomiya, Mari Kannagi

**Affiliations:** 1 Department of Immunotherapeutics, Graduate School of Medical and Dental Sciences, Tokyo Medical and Dental University, 1-5-45 Yushima, Bunkyo-ku, Tokyo, 113-8519, Japan; 2 Research Fellow of the Japan Society for Promotion of Science, Chiyoda-ku, Tokyo, 102-8472, Japan; 3 Department of Immunology, Graduate School of Medicine, University of the Ryukyus, Uehara, Okinawa, 903-0215, Japan; 4 Department of Hematology, Imamura Bun-in Hospital, Kagoshima, Kagoshima, 890-0064, Japan

**Keywords:** ATL, HTLV-1, IFN-α, PKR, Innate immunity, Anti-viral therapy, AZT, p53

## Abstract

**Background:**

Human T-cell leukemia virus type-1 (HTLV-1) is the causative retrovirus of adult T-cell leukemia/lymphoma (ATL) and HTLV-1-associated myelopathy/tropical spastic paraparesis (HAM/TSP). HTLV-1 gene expression is maintained at low levels *in vivo* by unknown mechanisms. A combination therapy of interferon-α (IFN-α) and zidovudin (AZT) shows therapeutic effects in ATL patients, although its mechanism is also obscure. We previously found that viral gene expression in IL-2-dependent HTLV-1-infected T-cells (ILTs) derived from ATL patients was markedly suppressed by stromal cells through a type I IFN response. Here, we investigated the effects of IFN-α with or without AZT on viral gene expression and cell growth in ILTs.

**Results:**

ILTs expressed variable but lower amounts of HTLV-1 Tax protein than HTLV-1-transformed HUT102 cells. Following the addition of IFN-α, the amounts of HTLV-1 p19 in the supernatants of these cells decreased in three days, while HTLV-1 gene expression decreased only in ILTs but not HUT102 cells. IFN-α also suppressed the spontaneous HTLV-1 induction in primary ATL cells cultured for 24 h. A time course study using ILTs revealed that the levels of intracellular Tax proteins decreased in the first 24 h after addition of IFN-α, before the reduction in HTLV-1 mRNA levels. The initial decreases of Tax protein following IFN-α treatment were observed in 6 of 7 ILT lines tested, although the reduction rates varied among ILT lines. An RNA-dependent protein kinase (PKR)-inhibitor reversed IFN-mediated suppression of Tax in ILTs. IFN-α also induced cell cycle arrest at the G0/G1 phase and suppressed NF-κB activities in these cells. AZT alone did not affect HTLV-1 gene expression, cell viability or NF-κB activities. AZT combined with IFN-α markedly induced cell apoptosis associated with phosphorylation of p53 and induction of p53-responsive genes in ILTs.

**Conclusions:**

IFN-α suppressed HTLV-1 gene expression at least through a PKR-mediated mechanism, and also induced cell cycle arrest in ILTs. In combination with AZT, IFN-α further induced p53 signaling and cell apoptosis in these cells. These findings suggest that HTLV-1-infected cells at an IL-2-dependent stage retain susceptibility to type I IFN-mediated regulation of viral expression, and partly explain how AZT/IFN-α produces therapeutic effects in ATL.

## Background

Human T-cell leukemia virus type 1 (HTLV-1) causes adult T-cell leukemia/lymphoma (ATL) [1-3], a malignant lympho-proliferative disorder resistant to chemotherapy. The virus is also responsible for HTLV-1-associated myelopathy/tropical spastic paraparesis (HAM/TSP) [4,5], a chronic inflammatory demyelinating disorder. Despite such severe clinical outcomes, levels of HTLV-1 gene expression are thought to be very low *in vivo*. HTLV-1 mRNA, but not proteins, are detectable in peripheral blood mononuclear cells (PBMCs) of HTLV-1-infected individuals [6]. Although undetectable, a low level of HTLV-1 proteins must be present *in vivo*, as HTLV-1-infected individuals maintain antibodies against HTLV-1 structural proteins and Tax protein-specific cytotoxic T lymphocytes.

Recent therapeutic approaches, such as allogeneic hematopoietic stem cell transplantation (allo-HSCT) [7,8], a humanized antibody therapy targeting CCR4 [9,10], or anti-viral therapy with interferon (IFN)-α and zidovudin (AZT) [11-13] partly improved ATL prognosis. *Ex vivo* studies have indicated that graft-versus-tumor responses including anti-Tax cytotoxic T-cells were potentially involved in the therapeutic mechanisms of allo-HSCT [14], and that the CCR4-antibodies were capable of inducing antibody-dependent cellular cytotoxicities [15]. However, combining AZT/IFN-α hardly affects HTLV-1-infected cells *in vitro*[16], and the mechanisms of its therapeutic effects remain unclear. A recent report indicated that the triple combination of arsenic trioxide/ IFN-α/AZT demonstrated more favorable therapeutic effects in ATL patients [17]. The combination of arsenic trioxide and IFN-α has been reported to induce proteolysis of Tax in HTLV-1-infected cells *in vitro*[18,19]. As IFN-α is indispensable in AZT/IFN-α, arsenic trioxide/IFN-α or arsenic trioxide/IFN-α/AZT therapies, ATL cells might be susceptible to IFNs *in vivo*.

It is well established that HTLV-1-infected cells are resistant to type I IFNs *in vitro*. For example, IFN-α reduced the virus release but not viral protein synthesis in HTLV-1-transformed HUT102 or MT-2 cells [20]. The mechanisms of the resistance to type I IFNs in HTLV-1-infected cells include reduction in the phosphorylation of Tyk2 and STAT2 [21], Tax-mediated competition with CREB binding protein/p300 [22], Tax-mediated up-regulation of SOCS1 [23,24], and up-regulation of IRF4 [25], all of which result in inhibition of IFN signaling. This may explain why IFN-α combined with AZT does not affect HTLV-1-infected cells *in vitro*, while conflicting with the clinical effects of AZT/IFN-α therapy in ATL patients. This discrepancy between *in vivo* and *in vitro* systems can be partially attributed to differences in status of HTLV-1-infected cells between the two systems.

We previously found that HTLV-1-infected cells could induce type I IFN responses in co-cultured stromal cells [26]. We also found that viral expression in HTLV-1-infected T-cells is markedly suppressed at both mRNA and protein levels through type I IFN responses mediated by stromal cells co-cultured [26]. This observation again conflicts with the previous notion of HTLV-1-mediated resistance to type I IFNs *in vitro.* Our experimental system differed from previous studies in two ways. First, we used IL-2-dependent HTLV-1-infected T-cells (ILTs) derived from ATL patients, while previous studies used IL-2-independent HTLV-1-transformed cell lines such as HUT102. Second, we used stromal cells as effectors; these mediated the type I IFN response, but could have also produced multiple factors other than IFNs.

In the present study, we investigated whether purified type I-IFNs can affect viral expression and cell growth of HTLV-1-infected cells by using various ILTs. Here we report a novel finding that IFN-α suppresses intracellular Tax expression at a translational level at least through PKR. We further demonstrate that IFN-α activates p53 pathways in cooperation with AZT, partly explaining the mechanisms of the therapeutic effects of AZT/IFN-α in ATL.

## Results

### Effects of IFN-α on HTLV-1 p19 release and viral transcription

**Figure 1:**
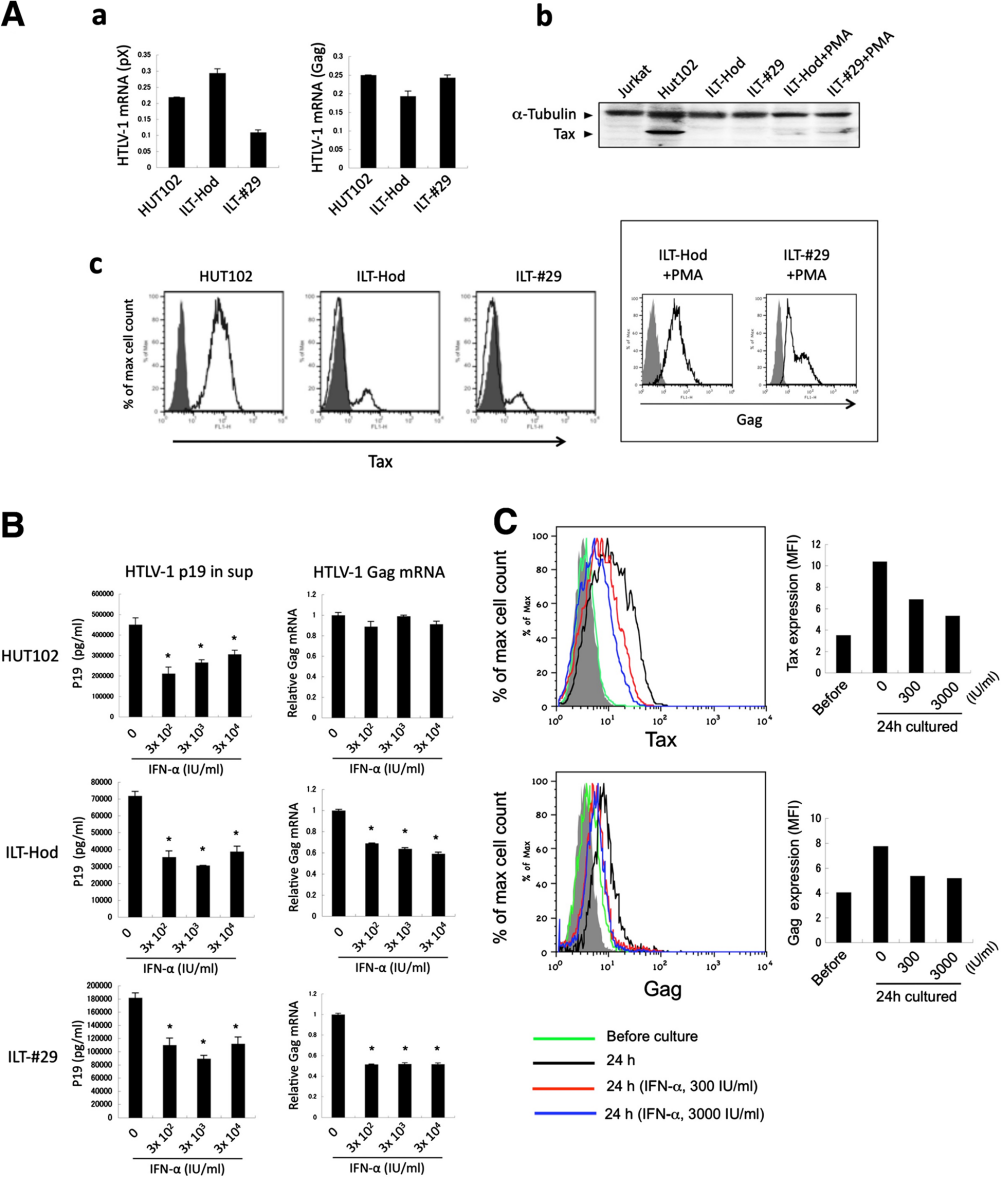
**Effects of IFN-α treatment on HTLV-1 p19 release and viral transcription in various HTLV-1-infected cell lines. A**. Expression of HTLV-1 mRNAs (**a**) and proteins (**b**, **c**) were evaluated by quantitative RT-PCR (**a**), immunoblotting (**b**), and flow cytometry (**c**), respectively, in HTLV-1-infected HUT102, ILT-Hod and ILT-#29 or uninfected Jurkat cell lines. **a**. The mRNA copy numbers measured by using pX or Gag primers were standardized to those for GAPDH and indicated as the means and standard deviations (SD) of duplicate samples. **b**. Cell lysates from indicated cell lines were subjected to an immunoblotting assay with antibodies to Tax (40 kDa) and α-Tubulin (50 kDa). The lysates in lanes 5 and 6 were prepared from ILT-Hod and ILT-#29 cells stimulated with PMA (50 ng/ml) overnight, respectively. **c**. Intracellular Tax proteins in permeabilized cells were stained with Alexa Fluor 488-labeled anti-Tax mAb (open histogram) and mouse IgG3 isotype control antibody (closed histogram). The inserted box indicates Gag expression in ILT-Hod and ILT-#29 cells stimulated with PMA (50 ng/ml) for 17h. **B**. HUT102 (top), ILT-Hod (middle) and ILT-#29 (bottom) cells were cultured for 3 days with or without three doses of IFN-α indicated. HTLV-1 p19 concentrations in the supernatants (left) and Gag mRNA levels were measured by ELISA and quantitative RT-PCR, respectively. Data are presented as the means and SD of duplicate samples. **C**. Frozen stored primary ATL cells were thawed and analyzed for intracellular Tax (top) or Gag (bottom) proteins by flow cytometry immediately (green line) or 24 h after culture with no (black line), 300 IU/ml (red line) or 3000 IU/ml (blue line) of IFN-α in the presence of IL-2 (30 IU/ml). The closed histogram represents samples stained with isotype controls. The mean fluorescence intensity (MFI) of each histogram was indicated in the bar graphs.

We evaluated the baseline levels of HTLV-1 gene expression in HUT102, ILT-Hod and ILT-#29 cell lines (Figure 1A). Relative levels of HTLV-1 mRNA in ILT-Hod and ILT-#29 cells were comparable with those in HUT102 cells. However, the levels of Tax protein in ILT-Hod and ILT-#29 cells were much lower than those of HUT102, and were barely detectable by immunoblotting only after stimulation of ILTs with phorbol 12-myristate 13-acetate (PMA). Flow cytometry results also indicated that ILT-Hod and ILT-#29 cells expressed smaller amounts of intracellular Tax protein than HUT102 cells. In addition, our analyses often identified Tax-negative cell populations in ILTs, with the ratio of these populations fluctuating during culture. These cells are also HTLV-1-infected, as all the cells in ILT-Hod and ILT-#29 cultures express HTLV-1 Gag protein after stimulation with PMA (Figure 1A insert), suggesting a dynamic turnover of HTLV-1 proteins in ILTs. Tax expression in HUT102 cells was apparently stable (Figure 1A).

We added IFN-α at various concentrations (300, 3000, and 30000 IU/ml) on HUT102, ILT-Hod, and ILT-#29 cells (Figure 1B). The amounts of HTLV-1 p19 released in supernatants significantly decreased after 72 h in culture for all the cell lines tested. Gag mRNA levels were also decreased in ILT-Hod and ILT-#29 in 3 days of culture (Figure 1B). These suppressive effects were observed at all doses of IFN-α used, indicating that 300 IU/ml of IFN-α was sufficient to produce these effects. In HUT102 cells, IFN-α suppressed HTLV-1 p19-release but not viral transcription, which is in agreement with previous reports [20].

We also examined the effects of IFN-α in primary ATL cells (Figure 1C). In the absence of IFN-α, intracellular expression of HTLV-1 proteins was spontaneously induced in ATL cells within 24 h after the initiation of culture. IFN-α suppressed the induction of Tax expression in these cells at a concentration of 3000 IU/ml more efficiently than 300 IU/ml. IFN-α also suppressed induction of Gag protein expression but equally at two doses.

Because HTLV-1 mRNA expression was suppressed in ILT-Hod and ILT-#29 cells as well as primary ATL cells following IFN-α treatment, we used these ILTs for further study on the effects of IFN-α at a dose of 3000 IU/ml hereafter.

### IFN-α reduced Tax protein expression before reduction of pX mRNA

**Figure 2:**
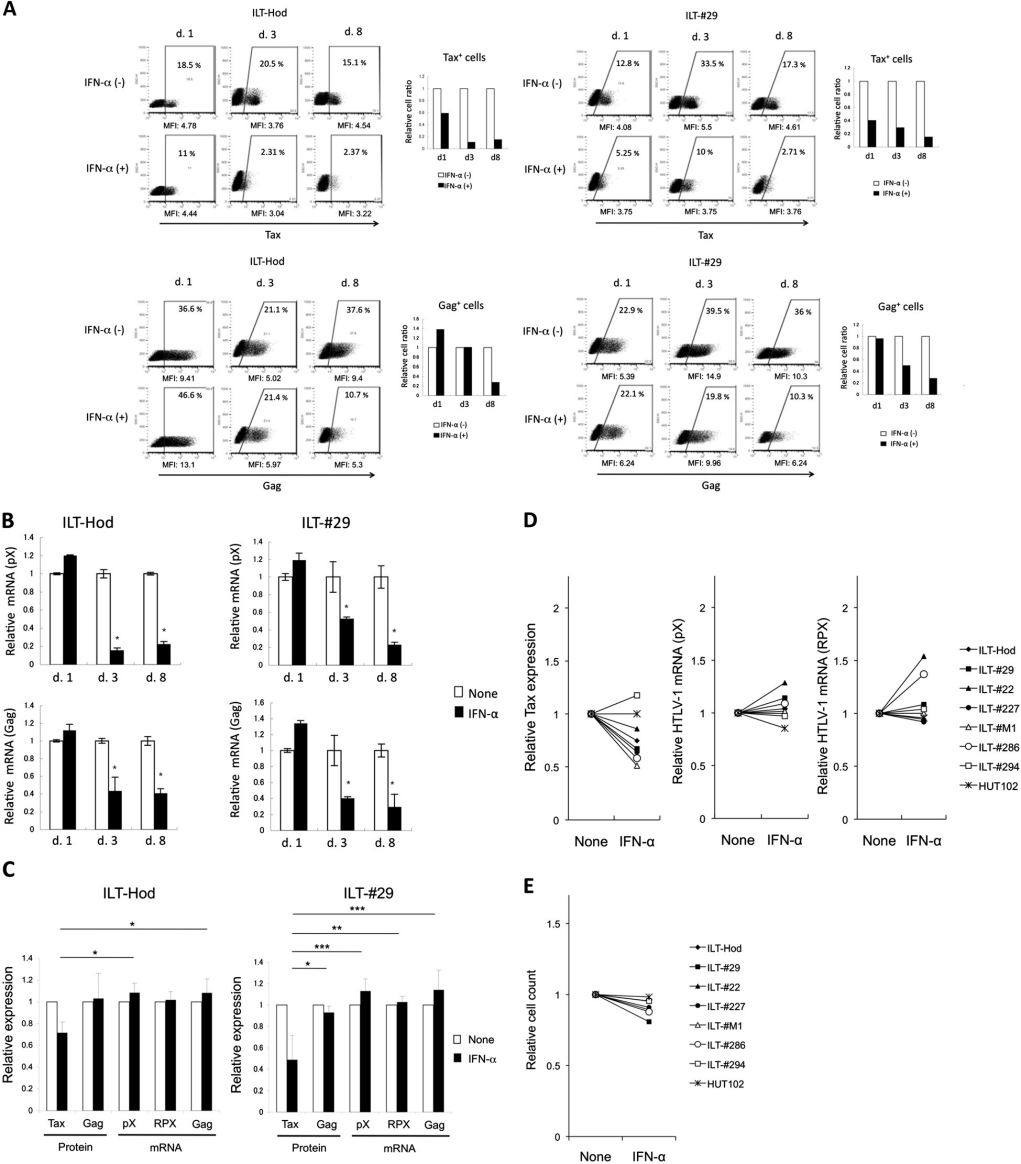
**IFN-α suppressed Tax protein expression before an apparent reduction in HTLV-1 mRNA levels. A**. The effects of IFN-α (3000 IU/ml) on intracellular Tax (top) and Gag (bottom) protein expression in ILT-Hod (left) and ILT-#29 (right) cells was evaluated by flow cytometry on days 1, 3, and 8 of culture. Cells stained with isotype antibodies served as negative controls. The values inside the dot plots represent percentages of viral protein-expressing cells, and the relative values in IFN-α-treated (closed bar) against untreated (open bar) samples are shown in the bar graph. The MFI value of the total cell population is indicated below the dot plots. **B**. Expression of HTLV-1 mRNA in the same cell samples prepared in A was evaluated by quantitative RT-PCR using pX (top) and Gag (bottom) primers. Results are standardized and presented as relative values of IFN-α-treated (closed bar) against untreated (open bar) samples. The means and SD of duplicate samples are indicated. *p < 0.05. **C**. HTLV-1 proteins (Tax and Gag) and HTLV-1 mRNAs expression in ILT-Hod and ILT-#29 cells were measured 24 h after incubation with (closed bar) or without (open bar) IFN-α, and the relative values were indicated as the means and SD of three independent experiments. Three different primer sets (pX, RPX, and Gag) were used to quantify HTLV-1 mRNAs. **D**. Seven ILT lines from various patients and HUT102 were cultured with or without IFN-α for 24 h, and the proportions of Tax positive cells (left) and the HTLV-1 mRNA quantified using pX (middle) and RPX (right) primers were indicated as relative values against the sample without IFN-α. **E**. Various HTLV-1-infected T cell lines shown in D were cultured with or without IFN-α for 3–4 days, and viable cell numbers analyzed by a colorimetric assay were indicated as relative values.

We next examined the time-course of IFN-α effects on Gag and Tax expression at protein and mRNA levels in ILT-Hod and ILT-#29 cells. Expression of intracellular Tax protein decreased within 1 day after addition of IFN-α to both cell lines. Intracellular Tax expression was maintained at lower levels than the control without IFN-α for at least 8 days (Figure 2A, top panels). Intracellular Gag protein expression in IFN-α-treated cells became lower than untreated cells at later time points (3–8 days), although the levels of viral expression fluctuated during culture (Figure 2A, bottom panels). Expression of HTLV-1 mRNAs in both cell lines were comparable to untreated cells or slightly increased in 1 day after IFN-α treatment, despite the reduction in Tax protein. At later time points (3–8 days), HTLV-1 mRNA levels were significantly decreased (Figure 2B). Thus, in IFN-α-treated ILTs, Tax protein was reduced first without apparent reduction in viral transcription, followed by reduction in viral mRNA and other viral protein expression.

We compared the levels of HTLV-1 proteins and mRNAs at 1 day after IFN-α treatment in these ILTs in several experiments, and confirmed that, at this time point, IFN-α reproducibly suppressed Tax protein levels in both cell lines, whereas the effects of IFN-α were inconsistent on Gag protein levels and not suppressive on HTLV-1 mRNA levels measured by using two different primer sets specific for pX and one for Gag regions (Figure 2C).

We further examined the effects of IFN-α on Tax protein and pX mRNA expression in several other ILT lines derived from ATL and HAM/TSP patients (Figure 2D). Although the suppression rates varied among cell lines, IFN-α suppressed intracellular Tax expression in 6 of 7 ILT cell lines tested in 24 h after IFN-α treatment. In ILT-#294 and HUT102 cells, Tax expression was not suppressed by IFN-α. HTLV-1 mRNA levels were not markedly suppressed or even enhanced in some cell lines in 24 h. Transient enhancement of HTLV-1 mRNA levels were sometimes observed also in ILT-Hod or ILT-#29 in 1 day after IFN-α treatment (Figure 2B, C). The effects of IFN-α on cell growth were limited, with mild reductions observed in some ILT lines after 3–4 days of culture (Figure 2E).

### PKR was involved in IFN-α-mediated reduction of Tax protein expression

**Figure 3:**
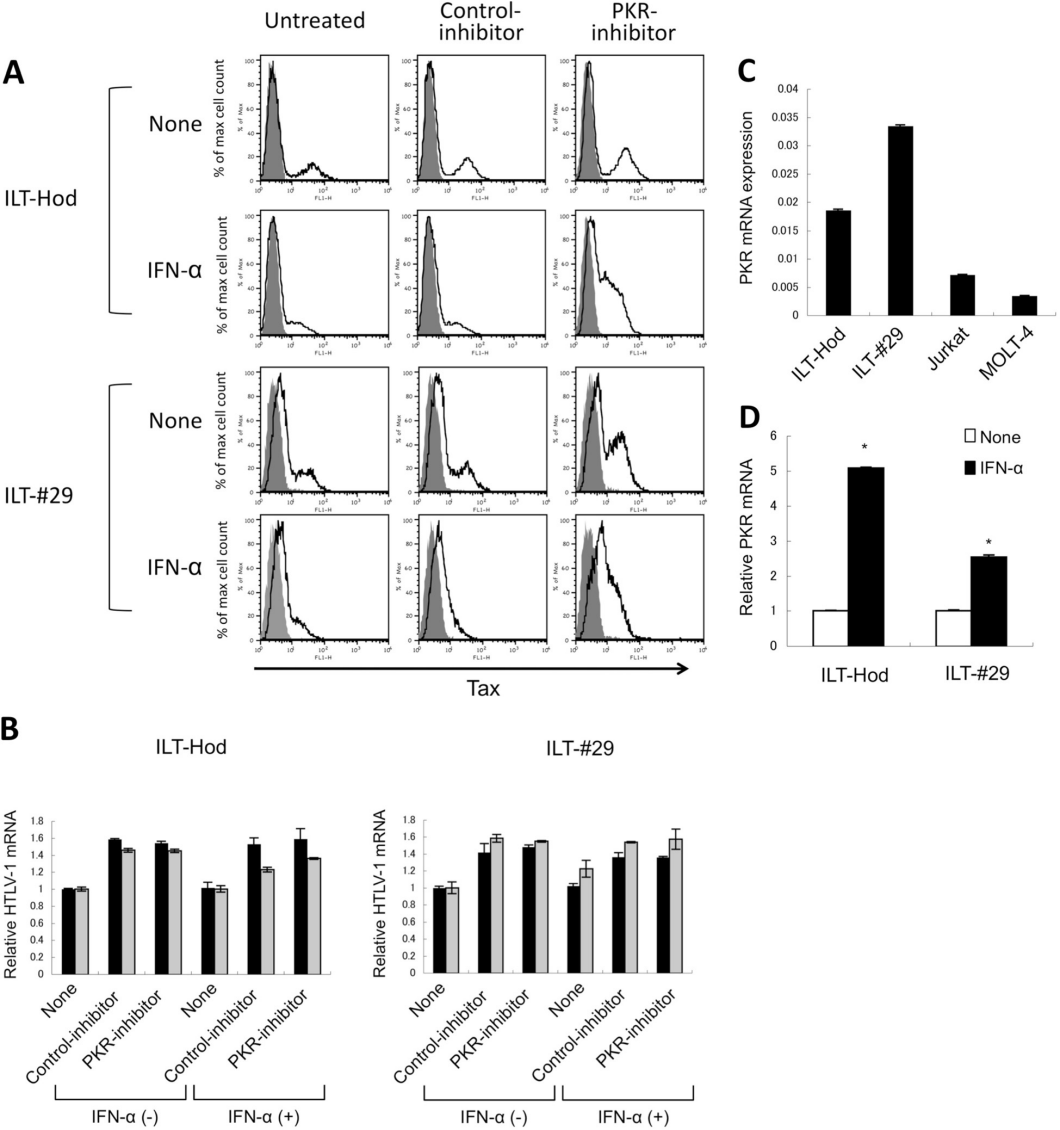
**Involvement of PKR in IFN-α-mediated reduction of Tax protein levels in HTLV-1-infected cells. A**. ILT-Hod and ILT-#29 cells were incubated with or without the PKR-inhibitor (500 nM) or the negative control-inhibitor (500 nM) for the first 2 h, then further cultured for the next 24 h in the presence or absence of IFN-α (3000 IU/ml) as indicated. Flow cytometry was then performed following stained with anti-Tax (open histogram) and isotype control (closed histogram) antibodies. **B**. HTLV-1 pX mRNAs in the same samples prepared in A were quantified by RT-PCR using two different primer sets (RPX; black bar, and pX; gray bar), standardized to GAPDH mRNAs, and the relative values were indicated as the means and SD of duplicate samples. **C**. PKR mRNAs in ILT-Hod, ILT-#29, and HTLV-1-negative Jurkat and MOLT4 cells were quantified by RT-PCR, standardized to GAPDH mRNA and indicated as the means and SD of duplicate samples. **D**. PKR mRNAs in ILT-Hod and ILT-#29 cells were quantified 24 h after culture in the absence (open bar) or presence (closed bar) of IFN-α (3000 IU/ml), and the relative values are indicated as the means and SD of duplicate samples. *p < 0.05.

Since the reduction in intracellular Tax protein levels was induced by IFN-α at an earlier stage than for mRNA in ILT cells, we assumed that some post-transcriptional mechanisms such as PKR-induced translational suppression might be involved. We therefore treated ILT-Hod and ILT-#29 cells with IFN-α in the presence of a chemical PKR-inhibitor or its negative-control (Figure 3A). The otherwise decreased levels of Tax protein in both ILTs in the presence of IFN-α were markedly augmented by the PKR-inhibitor. In both ILTs, the negative-control inhibitor did not alter the Tax protein levels. Interestingly, the PKR-inhibitor increased Tax expression in the absence of IFN-α as well especially in ILT-#29 cells (Figure 3A). The enhancement of Tax expression by PKR-inhibitor was not a result of transcriptional regulation, as HTLV-1 mRNA levels in the cells treated with PKR-inhibitor were comparable to those with control inhibitor (Figure 3B).

We then assessed PKR mRNA expression in these cell lines (Figure 3C). Both ILT lines expressed higher levels of PKR mRNA than HTLV-1-negative Jurkat and MOLT4 cells (Figure 3C). Moreover, IFN-α treatment further increased PKR mRNA expression in ILTs (Figure 3D). These observations indicated that IFN-α suppressed Tax expression at translational level via PKR in ILTs, and also suggested that similar mechanisms might regulate Tax expression in these cells to some extent without exogenous IFN-α.

### Effects of IFN-α and AZT on HTLV-1 expression and cell growth

Combination therapy with IFN-α and AZT has been reported to achieve high response rates especially in patients with smoldering and chronic types of ATL, although patients with acute type ATL frequently relapse after therapy [13]. Despite favorable clinical responses, the combination of IFN-α and AZT reportedly shows minimal effects on the viability of HTLV-1-transformed T cells *in vitro*[16]. As we found that IFN-α affected viral expression in ILT-Hod and ILT-#29 cells in our system, we then examined the effects of IFN-α and AZT using these ILTs.

**Figure 4:**
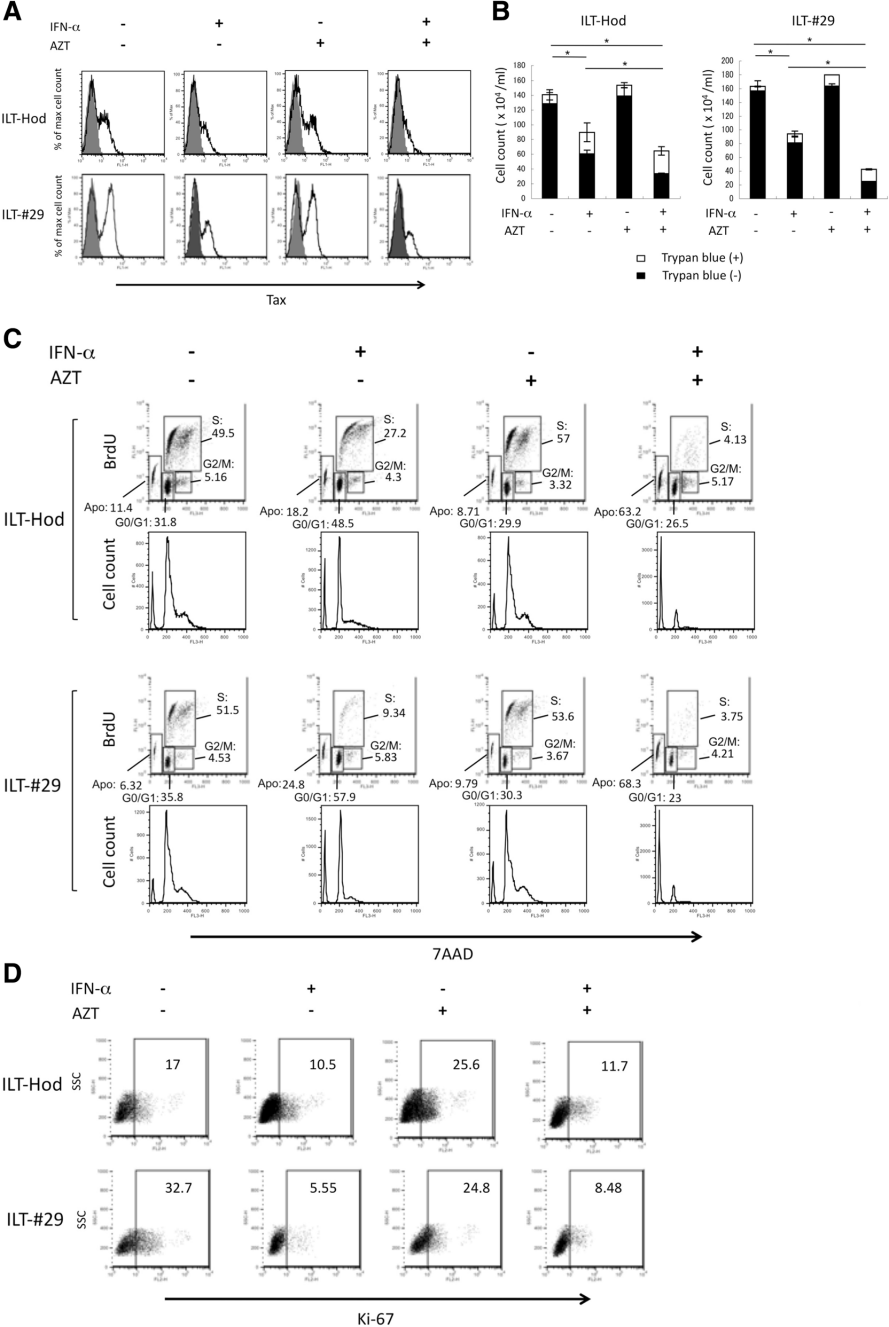
**Effects of IFN-α and AZT on HTLV-1 expression and cell growth of HTLV-1 infected cells.** ILT-Hod and ILT-#29 cells (10^6^/ml) were cultured in the absence or presence of IFN-α (3000 IU/ml) and/or AZT (10 μM) as indicated, and HTLV-1 expression (**A**), cell growth (**B**), cell cycle (**C**), and Ki-67 expression (**D**) in the cells were evaluated. **A**. Expression of intracellular Tax protein 3 days after the initiation of culture was evaluated by flow cytometry following stained with anti-Tax (open histogram) and isotype control (closed histogram) antibodies. **B**. ILT-Hod and ILT-#29 cells were similarly treated with IFN-α and/or AZT, and maintained with addition of equal volumes of fresh medium without IFN-α or AZT on the day 1 and 3, then viable (closed bar) and non-viable (open bar) cell numbers in cultures were evaluated by trypan blue exclusion on the day 8. *p < 0.05. **C**. ILT-Hod and ILT-#29 cells similarly treated with IFN-α and/or AZT were subjected to cell cycle analysis on the day 8. Cultures were treated with BrdU (10 μM) for the last 24 h of culture then permeabilized and incubated with a FITC-labeled mouse anti-BrdU antibody and 7AAD. Cells that are 7AAD-negative can be considered apoptotic (Apo). BrdU-negative and 7AAD-intermediate positive cells are in the G0/G1 phase. BrdU-positive and 7AAD-positive cells are in the S phase. BrdU-negative and 7AAD-highly positive cells are in the G2/M phase. The values in the dot plots indicate the proportion of the cells (%) in each phase. **D**. ILT-Hod and ILT-#29 cells similarly treated with IFN-α and/or AZT were analyzed for intracellular Ki-67 expression by flow cytometry on the day 8. The values in the dot plots indicate the proportion of Ki-67-positive cells (%).

The effects of these drugs on HTLV-1 expression in ILTs was first evaluated. After three days of incubation, when IFN-α-mediated suppression of intracellular Tax protein expression was clearly observed, similar levels of suppression were produced by treatment with the combination of IFN-α and AZT, but not with AZT alone (Figure 4A).

Next, we assessed the effects of these drugs on cell growth. Treatment of IFN-α alone induced mild suppression of cell propagation in one week of culture, while AZT alone did not. The combination of IFN-α and AZT showed stronger suppression of cell growth than IFN-α alone (Figure 4B). The cell cycle analysis indicated that cells treated with IFN-α alone, but not AZT alone, accumulated in the G0/G1 phase. Combined AZT/IFN-α showed a marked increase in apoptotic cell fractions in both ILT-Hod and ILT-#29 cells (Figure 4C). Expression of Ki-67 was also suppressed in these cells by treatment with IFN-α alone or AZT/IFN-α, but not with AZT alone (Figure 4D).

Therefore IFN-α, but not AZT, induced cell-cycle arrest and suppression of viral expression, while AZT combined with IFN-α induced apoptosis in ILT-Hod and ILT-#29 cells.

### Suppression of NF-κB activity by IFN-α treatment

**Figure 5:**
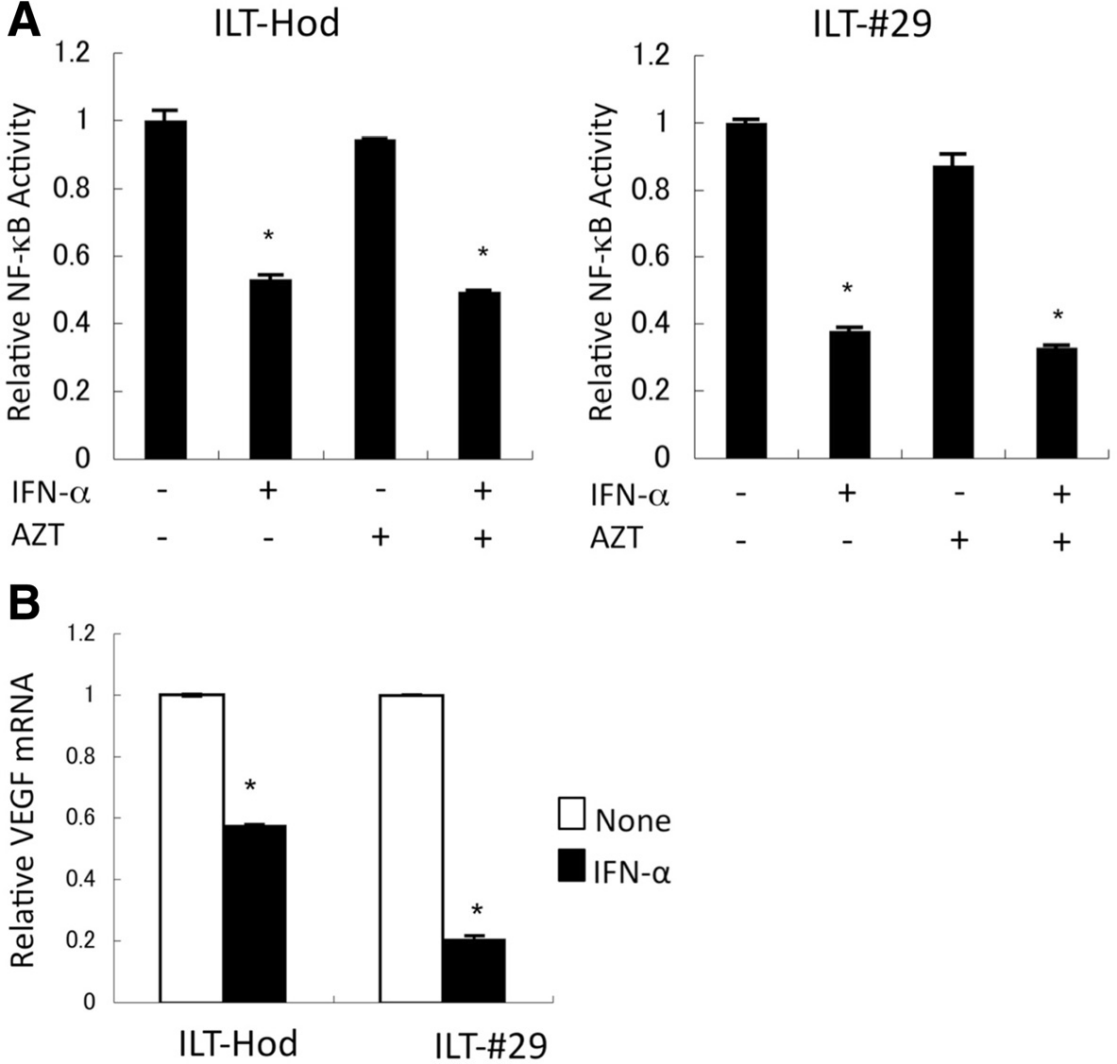
**Suppression of NF-κB activity by IFN-α in HTLV-1-infected cells. A**. ILT-Hod and ILT-#29 cells that were infected with lentiviral vectors containing reporter gene for the NF-κB responsive element and the TK-promoter several weeks before, were treated with or without IFN-α (3000 IU/ml) and/or AZT (10 μM) for 4 days as indicated. Luciferase activities were measured, and relative NF-κB activities normalized to TK-promoter activities were indicated as means and SD of duplicate samples. *p < 0.05. **B**. The levels of mRNA of VEGF, a NF-κB-regulated gene, in ILT-Hod and ILT-#29 cells 3 days after incubation with (closed bar) or without (open bar) IFN-α (3000 IU/ml) were quantified by RT-PCR and standardized to GAPDH mRNA. The relative values are indicated as means and SD of duplicate samples. *p < 0.05.

NF-κB pathway is constitutively activated and plays a critical role on cell survival in HTLV-1-infected, through Tax-mediated transactivation and other unknown mechanisms [27-29]. We examined the effects of AZT/IFN-α on NF-κB activity using ILT-Hod and ILT-#29 reporter cells stably expressing the NF-κB-responsive element reporter gene. In both cell lines, NF-κB activity was partly but significantly suppressed by IFN-α alone or in combination with AZT, but not with AZT alone (Figure 5A). The reduction in NF-κB activity by IFN-α was also confirmed by the decreases in the mRNA levels of vascular epithelial growth factor (VEGF), one of the NF-κB-regulated genes, in both ILTs treated with IFN-α (Figure 5B).

### Involvement of p53-signalling in IFN-α/AZT-mediated apoptosis in ILTs

**Figure 6:**
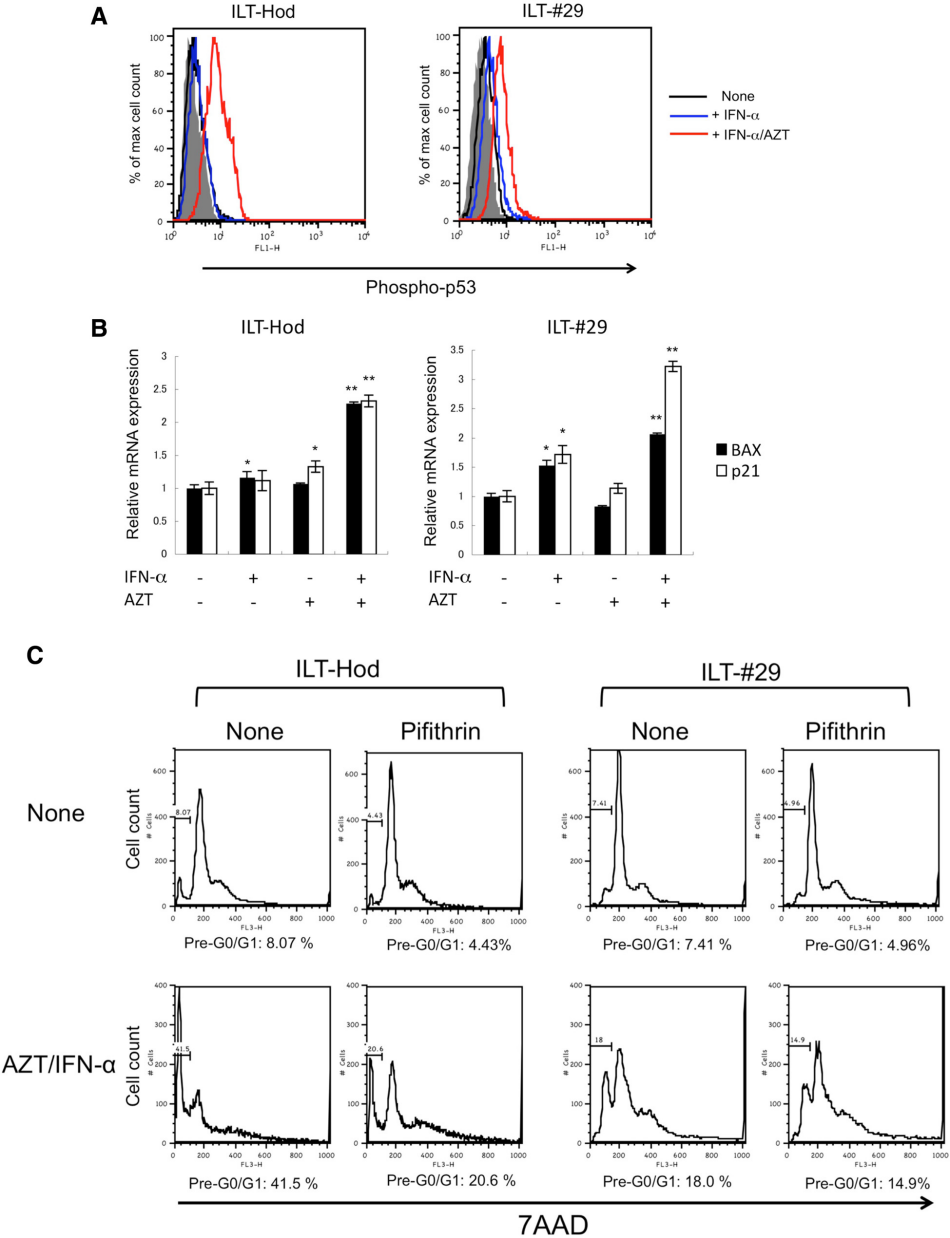
**Induction of p53-signaling by IFN-α and AZT in HTLV-1-infected cells. A**. Intracellular phosphorylated p53 levels in ILT-Hod and ILT-#29 cells were evaluated by flow cytomertry 3 and 4 days after incubation, respectively, in the absence (black line) or presence of IFN-α (3000 IU/ml) alone (blue line), or IFN-α/AZT (10 μM) (red line). The closed histograms indicate cells stained with control antibody. **B**. ILT-Hod and ILT-#29 were treated with IFN-α and/or AZT for 4 days and mRNA expression of BAX (closed bar) and p21 (open bar) was evaluated by quantitative RT-PCR. Results are standardized with the copy number of GAPDH mRNA, and the relative values are indicated as means and SD of duplicate samples. *p < 0.05, **p ≤ 0.01. **C**. ILT-Hod and ILT-#29 cells were cultured with or without IFN-α/AZT in the presence or absence of a p53-inhibitor (Pifithrin-α p-Nitro Cyclic, 1 μM) for 3 days and 5 days, respectively, then the cells were analyzed for the cell cycle by flow cytometry following 7AAD-staining. The proportions of apoptotic cell fractions (pre G0/G1) were indicated below each histogram.

We finally assessed the effect of IFN-α and AZT on p53 signaling that is known to be impaired in ATL cells [30]. We measured the phosphorylation of p53 in ILTs by flow cytometry (Figure 6A). The levels of phosphorylated p53 clearly increased in both ILTs following treatment with AZT/IFN-α, while IFN-α alone produced minimal effects.

We also evaluated the activity of the p53 pathway by measuring mRNA levels of p53-responsive genes, BAX and p21 (Figure 6B). Levels of BAX and p21 mRNAs were significantly increased in both cell lines treated with the combination of AZT and IFN-α. IFN-α alone slightly enhanced BAX and p21 mRNA levels in ILT-#29 cells but not in ILT-Hod cells. Effects of AZT alone were marginal in both cell lines.

The use of a p53-inhibitor partly reduced the apoptotic fraction in AZT/IFN-α-treated ILTs compared with those without inhibitor (Figure 6C). The effects of the p53-inhibitor were limited, however, probably because of a short half-life of the inhibitor.

These observations indicated that the combination of AZT and IFN-α effectively activated p53 pathway that was invoved in cell apoptosis in ILT-Hod and ILT-#29 cells.

## Discussion

In the present study, we have demonstrated that IFN-α suppressed HTLV-1 gene expression in infected cells. This is consistent with our previous findings, which indicated that stromal cells suppressed viral expression in HTLV-1-infected T-cells via type I IFN when co-cultured [26]. However, these findings conflict with most other reports [16,20-22]. Differences among opposing findings can be attributed to the differences in the HTLV-1-infected cells used. It has been reported that type I IFNs inhibit HTLV-1 p19 release but not viral gene expression in HTLV-1-transformed cells [20]. This was true for HUT102 cells also in the present study, but not for ILT cells (Figure 1B). One of the differences between HUT102 and ILTs is the levels of Tax protein, which is present at much higher levels in HUT102 than ILTs. Because expression of HTLV-1 proteins is barely detectable *in vivo*, we hypothesize that HTLV-1-infected cells in vivo might retain susceptibility to IFNs similarly to ILTs rather than HUT102. Indeed, IFN-α suppressed HTLV-1 gene expression in primary ATL cells that was induced in a short-term culture *in vitro* (Figure 1C).

Reduction in intracellular Tax protein levels preceded transcriptional suppression of viral mRNA in ILTs when treated with IFN-α (Figure 2), indicating involvement of some post-transcriptional mechanisms such as decreased protein translation and/or increased proteolysis [19]. In this study, we found that PKR was involved in IFN-α–mediated Tax suppression (Figure 3). PKR is a ubiquitously expressed serine/threonine kinase, induced by IFNs and activated by double-stranded RNA to phosphorylate its substrates. These substrates include the alpha subunit of translation initiating factor elF-2, thereby resulting in inhibition of protein synthesis [31-33]. Since the Tax protein positively regulates HTLV-1 transcription through interaction with the HTLV-1 long terminal repeat (LTR) [34,35], it would be reasonable that suppression of HTLV-1 transcription followed the reduction in Tax protein levels. However, the PKR-mediated translational control alone does not explain why Tax protein decreased earlier than Gag protein following IFN-α treatment in ILTs (Figure 2A, C), suggesting the involvement of additional mechanisms to produce preferential reduction of Tax.

It is intriguing that ILTs often show a histogram with two phases in the flow cytometric analysis for HTLV-1 proteins especially for Tax, despite the fact that all the ILT cells are infected with HTLV-1. This suggests that Tax protein levels in ILTs fluctuate between detectable and undetectable levels during culture. For the HUT102 cells, there was always a single peak of Tax-positive cells (Figure 1A). Nevertheless, the HTLV-1 transcription levels are comparable in ILTs and HUT102 (Figure 1A). In addition, the PKR inhibitor abrogated IFN-α-mediated suppression of Tax expression in ILTs without changing mRNA levels (Figure 3A, B). We also found that addition of the PKR inhibitor enhanced Tax expression in the absence of exogenous IFN-α especially for ILT-#29 cells (Figure 3A). Moreover, PKR expression was spontaneously increased in ILTs and further augmented by IFN-α (Figure 3C, D). These findings suggest that Tax protein synthesis might be spontaneously regulated by PKR to some extent in these cells, although it is unclear what activates PKR. If highly structured transcripts from HTLV-1 themselves were the activators of PKR, they might also activate other molecules such as 2', 5'-oligoadenylate synthetase that can also suppress viral expression. HTLV-1 expression might be regulated by such negative feedback systems to maintain equilibrium levels in ILT cells. Further studies will be required to understand the entire system regulating HTLV-1 expression in infected cells.

We noticed some differences with respect to the effects of IFN-α on HTLV-1 gene expression, p19 release, and cell growth in various HTLV-1-infected cell lines, which cannot be fully explained simply by the different levels of Tax expression in these cells, implying the presence of multiple mechanisms resisting against signaling pathways downstream of the IFN-αβ receptor. The mechanisms other than Tax determining IFN susceptibility remain to be clarified.

NF-κB is activated in HTLV-1-infected cells and plays a critical role in survival of these cells [36]. Our results indicate that IFN-α suppressed both viral expression and NF-κB activity; AZT did not affect either of these. Because Tax is a strong activator of NF-κB, IFN-α-mediated reduction of Tax protein levels likely results in IFN-α-mediated suppression of NF-κB (Figure 5A). However, the suppression of NF-κB activity by IFN-α in ILTs was partial. This is presumably attributed to the incompleteness of IFN-α-mediated suppression of Tax expression, and also to the presence of Tax-independent mechanisms for NF-κB activation in these cells.

Although IFN-α inhibited cell growth in ILTs, it was not cytocidal. Cell cycle analysis revealed that IFN-α induced cell cycle arrest at G0/G1, indicating that IFN-α has only a static effect. Cell apoptosis increased when both AZT and IFN-α were added (Figure 4). It has been reported that type I IFNs induce expression of p53, but do not directly activate it [37]. The p53 transcription factor is activated by various stresses, and mediates cell cycle arrest or apoptosis through induction of many p53-regulated genes [38,39]. HTLV-1-infected cells, including ATL cells, mostly have intact p53 genes, expressing enhanced levels of p53, but its function is impaired [30,40]. Tax can inhibit the functions of p53 through various mechanisms including competition over the co-activator CBP/p300 that is required for trans-activation [27,41,42]. In the present study, we demonstrated that phosphorylation of p53 and expression of the p53-regulated genes (Bax and p21) were markedly enhanced by the presence of both AZT and IFN-α, while IFN-α alone exhibited marginal effects (Figure 6A, B). As exogenous IFN-α reduced Tax protein levels in ILTs (Figures 2A, 4A), it might unlock the Tax-mediated interference on p53 functions and enabled to activate p53-pathway following incorporation of AZT. This is consistent with clinical findings that AZT/IFN therapy is effective on ATL cases without mutations in p53 gene [43].

## Conclusions

In conclusion, we have demonstrated that IFN-α can suppress HTLV-1 gene expression in IL-2-dependent HTLV-1-infected cells, and that PKR plays a critical role in the suppression. We further demonstrated that IFN-α and AZT cooperate to activate the p53 pathway and induce apoptosis. Our findings have elucidated previously unknown mechanisms regarding the regulation of HTLV-1 expression in infected cells, and partially explain how the combination of AZT and IFN-α produces therapeutic effects in ATL.

## Methods

### Cells

The various ILT lines derived from ATL patients (ILT-Hod, ILT-#29, ILT-#22, and ILT-#227) or HAM/TSP patients (ILT-M1, ILT-#286, and ILT-#294) were maintained in RPMI 1640 medium (Life Technologies, Inc., Grand Island, NY) containing 10% fetal calf serum (FCS; Sigma Aldrich, St. Louis, MO), Antibiotic Antimycotic Solution (Sigma Aldrich) and 30–100 IU/ml of recombinant human IL-2 (Shionogi, Osaka, Japan). The IL-2-independent HTLV-1-infected T-cell line, HUT102, derived from a patient with mycosis fungoides [3], and uninfected T-cell lines, Jurkat [44] and MOLT4 [45] were also used. Mononuclear cells were isolated from the peripheral blood of an acute ATL patient under written informed consent, stored frozen in liquid nitrogen, and used as primary ATL cells for experiments immediately after thawing. This study was approved by the Institutional Review Board of the Tokyo Medical and Dental University.

### Antibodies

Alexa Fluor 488-conjugated Lt-4 [46], a mouse monoclonal antibody (mAb) against the HTLV-1 Tax protein, and Alexa Fluor 488 mouse IgG3, κ isotype control (Biolegend, SanDiego, CA) were used for detecting Tax. Mouse ascites containing GIN-7 [47], a mAb against the HTLV-1 p19 Gag protein, and control mouse ascites were used for detecting Gag together with Fluorescein-isothiocyanate (FITC)-conjugated goat anti-mouse immunoglobulin G (IgG) plus IgM (IgG + IgM) antibodies (KPL, Gaithersburg, MD) as secondary antibody. R-phycoerythrin (R-PE)-conjugated anti-human Ki-67 mouse mAb (BD Pharmingen), Alexa Fluor 488-conjugated rabbit anti-human phosphorylated p53 (Ser15) Ab (Beckman Coulter, CA), and their isotype controls were also used.

### Reagents

Natural type human IFN-α (Sumiferon; Dainippon Sumitomo Pharma, Osaka, Japan) was added to cell cultures at various concentrations. Zidovudin (AZT) (Retrovir; GlaxoSmithKline; Research Triangle Park, NC) was used at 10 μM, a concentration inhibiting reverse transcription without cell toxicity [48]. When culturing these cells for longer than 3 days, fresh medium without these reagents was added during culture for maintenance. A chemical PKR-inhibitor (C_13_H_8_N_4_OS; Calbiochem) and its negative control inhibitor (C_15_H_8_C_l3_NO; Calbiochem) were dissolved in DMSO and added at 500 nM in culture 2 h before IFN-α treatment and carried over through the culture. Pifithrin-α p-nitro cyclic, a chemical p53-inhibitor (Calbiochem), was used at 1 μM.

### Quantitative RT-PCR and primers

Aliquots (0.5 μg) of total RNA extracted from cells using Isogen (Nippon Gene, Tokyo, Japan) were treated with DNase (Ambion; Austin, TX) and subjected to reverse transcription (RT) with oligo(dT)20 primers followed by PCR using THUNDERBIRD qPCR Mix (Toyobo, Osaka, Japan). To quantify HTLV-1 mRNAs, three primer sets were used; Gag primers (forward, 5′-CCT TAC CAC GCC TTC GTA GAA CGC CTC AAC ATA GC-3′; reverse, 5′-TTT GTC TTT GGG GGT CCA GGT CTG ACA AGC CCG CA-3′) located at Gag region, pX primers (forward, 5′-CGG ATA CCC AGT CTA CGT GTT TGG AGA CT-3′; reverse, 5′-GAG CCG ATA ACG CGT CCA TCG ATG GGG TCC-3′) located at pX region, and RPX primers (forward, 5′-ATC CCG TGG AGA CTC CTC AA-3′; reverse, 5′-AAC ACG TAG ACT GGG TAT CC-3′) located at upstream and downstream of the second splice junction site of tax/rex mRNA [6]. The primers specific for PKR (forward, 5′-CCT GTC CTC TGG TTC TTT TGC T-3′; reverse, 5′-GAT GAT TCA GAA GCG AGT GTG C-3′), VEGF (forward, 5′-GGA GGG CAG AAT CAT CAC G-3′; reverse, 5′-TCG ATT GGA TGG CAG TAG CT-3′), BAX (forward, 5′-GAT GCG TCC ACC AAG AAG CT-3′; reverse, 5′-CGG CCC CAG TTG AAG TTG-3′), p21 (forward, 5′-CCA TGT GGA CCT GTC ACT GT-3′; reverse, 5′-TGG TAG AAA TCT GTC ATG CTG GTC-3′), and GAPDH (forward, 5′-TGA TTT TGG AGG GAT CTC GCT CCT GGA AGA-3′; reverse, 5′-GTG AAG GTC GGA GTC AAC GGA TTT GGT CGT-3′) were also used. The thermal cycling protocol involved an initial denaturation at 95°C for 30 s, then 40 cycles of denaturation at 95°C for 5 s, annealing and extension at 60°C for 30 s, and then detection of fluorescence from SYBR Green. Products were quantified and standardized against GAPDH mRNA copy numbers.

### Flow cytometry

For intracellular staining of HTLV-1 antigens, cells were fixed with 4% paraformaldehyde for 10 min and permeabilized with 100% methanol for 10 min on ice. To detect the Gag protein, cells were serially incubated with GIN-7 or control ascites and FITC-conjugated goat anti-mouse IgG + IgM. To detect the Tax protein, cells were incubated with Alexa Fluor 488-labelled Lt-4 or isotype control antibody. To stain intracellular Ki-67, cells were fixed with 4% paraformaldehyde and permeabilized with a BD Perm/Wash™ Buffer Kit (BD Pharmingen), then incubated with an R-PE-conjugated anti-human Ki-67 mouse mAb or isotype control mouse IgG1, κ antibody. For staining intracellular phosphorylated p53, cells were fixed with 4% paraformaldehyde for 10 min and permeabilized with 100% methanol for 10 min on ice prior to incubation with antibody. Stained cells were analyzed with a flow cytometer (FACSCalibur; Becton Dickinson, San Jose, CA) using FlowJo software (Tree Star).

### Immunoblotting

HTLV-1-infected or uninfected cells were dissolved in Cell Culture Lysis Reagent (Promega, Madison, WI) containing 25 mM Tris-phosphate (pH 7.8), 2 mM DTT, 2 mM 1,2-diaminocyclohexane-N,N,N',N'-tetraacetic acid, 10% glycerol, and 1% Triton X-100, with protease inhibitor cocktail (Roche Diagnostics, Basel, Switzerland), and were incubated on ice for 1 hour. The cell lysates were cleared by centrifugation, denatured with SDS sample buffer (Thermo Scientific, Rockford, IL) and 2.5% 2-mercaptethanol (Sigma-Aldrich, St.Louis, MD) at 70°C for 15 min, and 17 μg of proteins were electrophoresed on polyacrylamide gel (Oriental Instruments CO., LTD, Kanagawa, Japan), and then transferred to PVDF membrane (ATTO, Tokyo, Japan). The membranes was blocked with Block Ace (DS Pharma Biomedical Co., Ltd, Osaka, Japan) overnight, and reacted with mouse anti-Tax and anti-α-Tubulin (Cedarlane, Ontario, Canada) antibodies as primary antibodies overnight, followed by exposure to horseradish peroxidase-conjugated sheep anti-mouse IgG whole antibody (GE Healthcare, Pittsburgh, PA) as a second antibody. The reacted bands were visualized by enhanced chemiluminescence using Novel® ECL (Invitrogen, Carlsbad, CA) and analyzed on Image Quant mini LAS 4000 (GE Healthcare).

### Cell cycle analysis

Cells were cultured in the presence of 10 μM bromodeoxyuridine (BrdU) for 24 h, fixed and then permeabilized, followed by incubation with a mouse anti-BrdU mAb and 7AAD from BrdU flow Kits (BD Pharmingen), according to the manufacturers’ instructions. Stained cells were analyzed with a flow cytometer using CellQuest software (Becton Dickinson). To evaluate cell growth, Trypan blue exclusion test and a colorimetric assay using Cell Counting Kit-8 (Dojindo, Kumamoto, Japan) based on formazan color development were used.

### Enzyme-linked immunosorbent assays (ELISAs)

The concentration of HTLV-1 p19 in the supernatants from ILT-Hod, ILT-#29 or HUT102 cultures were measured using a RETRO-tek HTLV-1/II p19 antigen ELISA (ZeptoMetrix Corp., Buffalo, NY) according to the manufacturer’s instructions.

### Reporter assays

Reporter cell lines (ILT-Hod/NF-κB-Luc and ILT-#29/NF-κB-Luc) were established by using a Cignal Lenti-NF-κB reporter Luc Kit (Qiagen, Duesseldorf, Germany) and Cignal Lenti thymidine kinase (TK)-Renilla control (Qiagen). Luciferase assays were conducted with Luciferase or Renilla luciferase assay systems (Promega, Madison, WI) on cell lysates in Renilla luciferase lysis buffer (Promega). Relative NF-κB activity was calculated as the ratio of firefly luciferase to renilla luciferase activities in the same sample.

### Statistics

The unpaired *t*-test was performed for statistical significance, and *P* values less than 0.05 were considered significant.

## Abbreviations

ATL: Adult T-cell leukemia/lymphoma; AZT: Zidovudin (3'-Azido-3'-deoxythymidine); BrdU: Bromodeoxyuridine; ELISA: Enzyme-linked immunosorbent assay; FCS: Fetal calf serum; FITC: Fluorescein-isothiocyanate; HAM/TSP: HTLV-1-associated myelopathy/tropical spastic paraparesis; HSCT: Hematopoietic stem cell transplantation; HTLV-1: Human T-cell leukemia virus type-1; IFN-α: Interferon-α; IgG: Immunoglobulin G; ILT: IL-2-dependent HTLV-1-infected T-cell; mAb: Monoclonal antibody; MFI: Mean fluorescence intensity; PBMC: Peripheral blood mononuclear cell; PKR: RNA-dependent protein kinase; PMA: Phorbol 12-myristate 13-acetate; RT: Reverse transcription; TK: Thymidine kinase; VEGF: Vascular endothelial growth factor

## Competing interests

The authors declare that they have no competing interests.

## Authors’ contributions

SK carried out most of the experiments, analyzed data, and wrote the manuscript; MKi and AT carried out certain aspects of the experiments; AH advised on flow cytometry analysis; AS advised on signaling analysis; TM advised on RT-PCR analysis; YT provided HTLV-1-specific monoclonal antibodies; AU provided clinical samples; MKa designed the study, analyzed data, and wrote the manuscript; all authors reviewed and approved the final manuscript.
